# 4107 Implementation and evaluation of a novel protocol that uses clinical biomarkers to promote early diagnosis and treatment of Neurodevelopmental Disabilities

**DOI:** 10.1017/cts.2020.237

**Published:** 2020-07-29

**Authors:** Tara Lynn Johnson, Sowmya Sivakumar, Namarta Kapil, Bittu Majmudar

**Affiliations:** 1 University of Arkansas for Medical Sciences

## Abstract

OBJECTIVES/GOALS: Our objective was to establish a new protocol to evaluate new biomarkers to detect Neurodevelopmental Disabilities (NDD) in high-risk infants. As early intervention results in better outcomes, our goal was to implement the protocol to promote earlier NDD diagnosis and referral for treatment. METHODS/STUDY POPULATION: We implemented a new protocol using the General Movement Assessment (GMA), Hammersmith Infant Neurological Examination (HINE), and Capute Scales to evaluate infants who were at high risk of NDD. To determine the success of our protocol with these biomarkers, we studied former premature infants who were evaluated in follow-up clinic from 10/1/2018-10/1/2019. We defined our primary and secondary outcomes as the ages of neurodevelopmental diagnoses and referral to early intervention services before and after implementation of the new protocol, respectively. Our hypotheses were that infants who were evaluated with these biomarkers would be diagnosed with NDD and be referred for treatment at younger ages than their counterparts. RESULTS/ANTICIPATED RESULTS: Approximately 120 patients were evaluated during the time period that was defined. About half were evaluated prior to implementing the GMA and HINE, and the remainder were evaluated using GMA and other developmental measures. We anticipate that infants who underwent GMA will be diagnosed with NDD and referred for therapies at a younger age than their counterparts. DISCUSSION/SIGNIFICANCE OF IMPACT: Through our translational research, we will transform the standard of care for high-risk infants by incorporating clinical biomarkers into day-to-day clinical care for these infants. Implementation of this novel protocol will promote the early diagnosis and referral to treatment for NDD.

